# The Industrial Organism *Corynebacterium glutamicum* Requires Mycothiol as Antioxidant to Resist Against Oxidative Stress in Bioreactor Cultivations

**DOI:** 10.3390/antiox9100969

**Published:** 2020-10-09

**Authors:** Fabian Stefan Franz Hartmann, Lina Clermont, Quach Ngoc Tung, Haike Antelmann, Gerd Michael Seibold

**Affiliations:** 1 Section for Synthetic Biology, Department of Biotechnology and Biomedicine, Technical University of Denmark, 2800 Kongens Lyngby, Denmark; fashart@dtu.dk; 2 Institute of Biochemistry, University of Cologne, 51149 Cologne, Germany; linaclermont@gmx.de; 3 Institute of Biology-Microbiology, Freie Universität Berlin, 10115–14199 Berlin, Germany; qtung@zedat.fu-berlin.de (Q.N.T.); haike.antelmann@fu-berlin.de (H.A.)

**Keywords:** *Corynebacterium glutamicum*, oxidative stress, mycothiol, Mrx1-roGFP2, redox potential

## Abstract

In aerobic environments, bacteria are exposed to reactive oxygen species (ROS). To avoid an excess of ROS, microorganisms are equipped with powerful enzymatic and non-enzymatic antioxidants. *Corynebacterium glutamicum*, a widely used industrial platform organism, uses mycothiol (MSH) as major low molecular weight (LMW) thiol and non-enzymatic antioxidant. In aerobic bioreactor cultivations, *C. glutamicum* becomes exposed to oxygen concentrations surpassing the air saturation, which are supposed to constitute a challenge for the intracellular MSH redox balance. In this study, the role of MSH was investigated at different oxygen levels (pO_2_) in bioreactor cultivations in *C. glutamicum*. Despite the presence of other highly efficient antioxidant systems, such as catalase, the MSH deficient Δ*mshC* mutant was impaired in growth in bioreactor experiments performed at pO_2_ values of 30%. At a pO_2_ level of 20%, this growth defect was abolished, indicating a high susceptibility of the MSH-deficient mutant towards elevated oxygen concentrations. Bioreactor experiments with *C. glutamicum* expressing the Mrx1-roGFP2 redox biosensor revealed a strong oxidative shift in the MSH redox potential (*E*_MSH_) at pO_2_ values above 20%. This indicates that the LMW thiol MSH is an essential antioxidant to maintain the robustness and industrial performance of *C. glutamicum* during aerobic fermentation processes.

## 1. Introduction

The Gram-positive soil bacterium *Corynebacterium glutamicum* is widely used as anindustrial workhorse primarily for the production of L-glutamate and L-lysine [1], and has been genetically engineered as a broad platform for production of several important industrial products [2,3]. Currently, *C. glutamiucm* is mostly used for aerobic production processes, but its facultative anaerobic metabolism allows to design efficient two-stage processes for the production of reduced chemicals including an aerobic growth phase and an anaerobic production phase [4,5].

Reactive oxygen species (ROS) are generated as an inescapable consequence of aerobic metabolism, caused by incomplete stepwise reduction of molecular oxygen during respiration [6,7]. ROS include superoxide anions (O_2_^−^), hydroxyl radicals (^•^OH), peroxy radicals (ROO^•^), alkoxy radicals (RO^•^), and hydrogen peroxide (H_2_O_2_) [6,7,8,9,10]. An excess of ROS leads to oxidative stress, which subsequently damages essential cell components, such as lipids, proteins, and nucleic acids [11]. To avoid irreversible damages, aerobic living organisms possess a wide range of ROS-scavengers including enzymatic and non-enzymatic detoxification systems [12,13,14,15,16,17].

Non-enzymatic antioxidant defense systems are represented by low molecular weight (LMW) thiols, which are essential to maintain a reducing environment in the cytoplasm [16]. Eukaryotes and most Gram-negative bacteria produce glutathione (GSH) as their major LMW thiol [18]. Bacteria of the Gram-positive phylum *Actinobacteria*, including *Mycobacterium tuberculosis*, *Mycobacterium smegmatis*, and *C. glutamicum*, however, utilize the GSH surrogate mycothiol (MSH) [19,20,21,22]. Upon the formation of ROS, the redox-active sulfhydryl group of MSH can either scavenge free radicals directly or function as a cofactor for antioxidant enzymes, resulting in the formation of oxidized mycothiol disulfide (MSSM) [14,15,23,24]. MSH can prevent overoxidation of protein thiols to sulfonic acids by forming mixed disulfides via a mechanism referred to as protein *S*-mycothiolation [25]. Irreversible overoxidation could cause a loss of cell viability and the requirement of the synthesis of new proteins in the case of essential and abundant proteins [25,26]. Upon treatment with hypochlorite, 25 *S*-mycothiolated proteins have been identified in *C. glutamicum*, indicating its protective function during oxidative stress [27]. Consequently, the absence of MSH was shown to increase oxidative stress sensitivity indicated by an oxidized environment, an impaired growth behavior, and a loss of cell viability [27,28,29].

Previous research regarding the physiological role of antioxidants in *C. glutamicum* exclusively used artificial oxidants to induce ROS generation rather than using conditions more relevant for production conditions such as bioreactor cultivations at different aeration rates [12,15,27,28,29]. Of note, ROS production rates are proportional to the collision frequency of oxygen and redox enzymes [7,8,9,30,31,32]. Especially during aerobic industrial fermentations, cells are exposed to oxygen concentrations often surpassing the air saturation. Hyperbaric oxygen was shown to be detrimental for growth patterns of various organisms, for example, *Escherichia coli*, *Bacillus subtilis*, and *Saccharomyces cerevisiae* [33,34,35]. *C. glutamicum*, as an aerobic industrial platform organism, is highly robust towards oscillations of low and high oxygen concentrations [36]. However, to the best of our knowledge, the contribution of the non-enzymatic antioxidant and main LMW thiol MSH in *C. glutamicum* to its robustness during aerobic fermentations has yet not been investigated. 

Here, we studied the relevance of the non-enzymatic antioxidant MSH in the industrial platform bacterium *C. glutamicum* during aerobic batch fermentations. Bioreactor experiments revealed an impaired growth behavior in the MSH deficient *C. glutamicum* Δ*mshC* mutant upon exposure to oxygen concentrations surpassing air saturation. Application of *C. glutamicum* strains expressing the stably integrated Mrx1-roGFP2 redox biosensor [29] enabled monitoring of the changes in the MSH redox potential (*E*_MSH_) in *C. glutamicum* WT during bioreactor cultivations at different oxygen levels. Altogether, the results of our study demonstrate the physiological importance of MSH as a non-enzymatic antioxidant in *C. glutamicum* to overcome oxidative stress during aerobic bioreactor cultivations.

## 2. Materials and Methods

### 2.1. Strains, Media, and Culture Conditions

The strains used in this study were *C. glutamicum* ATCC13032 (WT) [37], the MSH*-*deficient *C. glutamicum* ∆*mshC* deletion mutant [26], and the Mrx1-roGFP2 redox biosensor expressing strains *C. glutamicum* WT_Mrx1-roGFP2 and *C. glutamicum* ∆*mshC*_Mrx1-roGFP2 [29]. *C. glutamicum* strains were pre-cultured in 2xTY medium at 30 °C in 500 mL shake flasks. Prior to inoculation of the main-culture, cells of an overnight culture were washed twice with 100 mM potassium phosphate buffer (pH 7.0). *C. glutamicum* main cultures were grown in CGXII minimal medium [1] supplemented with 10 g L^−1^ or 20 g L^−1^ glucose as a carbon source for growth experiments in shake flasks and bioreactors, respectively.

Growth experiments in bioreactors were performed aerobically at 30 °C as 1 L cultures in 1.5 L jars in a BIOSTAT^®^ B fermentation system (Sartorius, Goettingen, Lower Saxony, Germany), as described previously [38]. The pH was maintained at 7.0 by online measurement using a standard pH electrode (Mettler Toledo, Giessen, Hessen, Germany) and addition of 4 M KOH and 4 M H_2_SO_4_. Partial oxygen pressure (pO_2_) was measured online using a polarimetric oxygen electrode (Mettler Toledo), and was adjusted to pO_2_ values provided in the text in a cascade by stirring at 200 to 800 rpm as well as by mixing N_2_ and air for the inlet gas. For anaerobic condition (pO_2_ = 0%), 100% N_2_ was used until a pO_2_ of 0% was reached. When required, AF204 antifoam agent (Sigma, MI, USA.) was added manually. The data were collected with the software MFCS (Sartorius BBI Systems, Goettingen, Lower Saxony, Germany). Growth in shake flasks and bioreactors was followed by measuring the optical density (OD 600_nm_).

### 2.2. Fluorescence Measurements of Mrx1-roGFP2 Biosensor Oxidation In Vitro and In Vivo

For testing suitable settings for fluorescence measurements of Mrx1-roGFP2 redox biosensor oxidation, *C. glutamicum* strains harboring genomic integrated Mrx1-roGFP2 [29] were pre-cultured in 2xTY medium until the stationary phase. For preparation of crude cell extracts, cells were harvested by centrifugation (4000 rpm, 10 min., 4 °C), washed twice in potassium phosphate buffer (100 mM, pH 7.0), and finally resuspended in 1 mL of the respective buffer solution. Disruption of the cells was conducted using a Ribolyzer (Precellys TM Control Device, Bertin Technologies, Montigny-le-Bretonneux, Department Yvelines, France) at 6000 rpm, four times for 30 s each. Cell debris were removed by centrifugation (12,000 rpm, 20 min; 4 °C) and 180 µL of the supernatant was transferred to black, flat-bottomed 96-well microplates (Thermo Fisher Scientific, Dreieich, Hessen, Germany) for further fluorescence analysis using a fluorescence spectrophotometer (SpectraMax iD3, Molecular Devices LLC, San Jose, CA, USA). After the addition of 20 µL oxidants (50 mM diamide), reductants (100 mM Dithiothreitol (DTT), and 100 mM potassium phosphate buffer for fully oxidized, fully reduced, and non-treated control samples, respectively, cells were incubated for 15 min at 30 °C as described previously [29]. Subsequently, excitation scans were conducted (360 nm–470 nm) by setting an emission wavelength of 510 nm. For in vivo fluorescence measurements, *C. glutamicum* strains expressing the Mrx1-roGFP2 biosensor were harvested by centrifugation (4000 rpm, 4 min) and washed in 100 mM potassium phosphate buffer (pH 7.0). Finally, an optical density of 40 was adjusted and 180 µL of the cell suspension was transferred to black, flat-bottomed 96-well microplates for fluorescence analysis. To determine the maximum and minimum oxidation ratios, 20 µL of different concentrated cumene hydroperoxide (CHP) and DTT solutions were added for oxidation and reduction of the biosensor probe, respectively, until the respective ratio reached its minimum and maximum value. For samples, 20 µL potassium phosphate buffer was added instead. Mrx1-roGFP2 fluorescence intensity was recorded at an emission intensity of 510 nm upon excitation at 380 nm and 470 nm. The corresponding biosensor oxidation degree (OxD) was calculated by normalizing to fully reduced as well as oxidized controls, as described previously [29,39,40], with the following Equation (1):

(1) $$\mathrm{OxD}=\frac{I380_{\mathrm{sample}}\times I470_{\mathrm{red}}-I380_{\mathrm{red}}\times I470_{\mathrm{sample}}}{I380_{\mathrm{sample}}\times I470_{\mathrm{red}}-I380_{\mathrm{sample}}\times I470_{\mathrm{ox}}+I380_{\mathrm{ox}}\times I470_{\mathrm{sample}}-I380_{\mathrm{red}}\times I470_{\mathrm{sample}}}$$

Here, *I*380_sample_ and *I*470_sample_ represent the measured fluorescence intensities received for an excitation at 380 nm and 470 nm, respectively. Fully reduced and oxidized controls are given by *I*380_red_, *I*470_red_ and *I*380_ox_, *I*470_ox_, respectively. Calculated OxD values; the standard midpoint redox potential of roGFP2 (*E*°`roGFP2 = −280 mV) [41]; Faraday constant (F: 96,485 C mol^−1^) of electric charge per mole of electrons, where 2 refers to the number of electrons transferred during the redox reaction; the respective temperature in Kelvin (T: 303.15 K); and the universal gas constant (R: 8.314 J K^−1^ mol^−1^) were used in order to determine the MSH redox potential (*E*_MSH_) via the Nernst Equation (2):

(2) $$E_{\mathrm{MSH}}=E_{\mathrm{roGFP}2}=E_{\mathrm{roGFP}2}^{o’}-\left.\left(\frac{\mathrm{RT}}{2F}\right.\right)\times \mathrm{In}\left.\left(\frac{1-\mathrm{OxD}}{\mathrm{OxD}}\right.\right)$$

### 2.3. Statistical Analysis

Analysis of one-way variance (ANOVA) with Tukey’s test was used to assess differences of biosensor oxidation degrees derived from *C. glutamicum* WT and MSH-deficient mutant strains harboring the genetically encoded biosensor Mrx1-roGFP2. Differences were considered statistically significant when *p* < 0.01.

## 3. Results

### 3.1. The MSH-Deficient Mutant is Susceptible to Elevated Oxygen Concentrations

**Figure 1:**
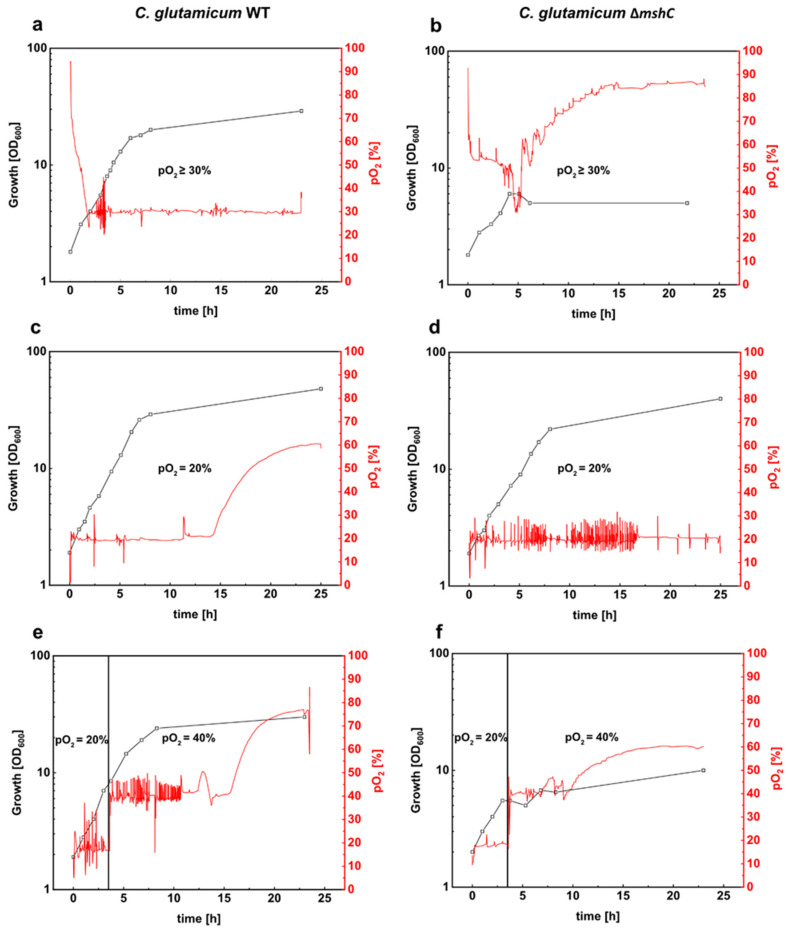
Batch cultivations of *C. glutamicum* WT (left plots) and the mycothiol-deficient *C. glutamicum* mutant ∆*mshC* (right plots) in stirred bioreactors. Both strains were cultivated in 1 L CgXII minimal medium (T = 30 °C, pH = 7.0; initial glucose concentration 20 g L^−1^). Bioreactor experiments were performed by setting different pO_2_ values. pO_2_ values of ≥30% regulated via stirring (200 rpm–800 rpm) (**a**,**b**). pO_2_ values of 20% were set by stirring as well as mixing nitrogen and air for the inlet gas (**c**,**d**). Finally, bioreactor experiments were carried out with an initial pO_2_ value of 20% during the first 3 h and a second fermentation phase with a pO_2_ value of 40% (**e**,**f**). Growth was monitored by measuring the optical density at 600 nm. Fermentations were performed in BIOSTAT^®^ B bioreactors. Data were collected with the software MFCS. OD, optical density.

To compare growth of *C. glutamicum* WT and the ∆*mshC* mutant, batch cultivations in stirred bioreactors were performed at a pO_2_ value of ≥30% (regulated in cascade via the stirring rate) and a constant pH of 7.0. These are common conditions used for production and physiological studies with *C. glutamicum* [42,43,44,45]. Growth of *C. glutamicum* WT proceeded with a rate of 0.37 h^−1^ and the cells reached a final OD_600_ of 29 after 24 h of cultivation (Figure 1a). Growth of the MSH-deficient ∆*mshC* mutant proceeded slower than *C. glutamicum* WT within the first hours of the bioreactor cultivation, resulting in a cessation of growth after 4 h (Figure 1b). During the course of cultivation with *C. glutamicum* WT, a stable pO_2_ value of 30% was reached after an expected initial phase with a higher pO_2_ (Figure 1a). For the MSH deficient strain, however, the decreased oxygen demand resulted in pO_2_ values always above the minimal pO_2_ of 30% (Figure 1b). This decreased oxygen demand of the ∆*mshC* mutant can be explained by the impaired growth during the first 4 h of cultivation. The rise of pO_2_ after 4 h of cultivation coincidences with the stop of growth of the MSH-deficient mutant (Figure 1b). Moreover, strong foam formation was observed at this phase of the bioreactor cultivation with the ∆*mshC* mutant. Taken together, the growth deficit of the ∆*mshC* mutant revealed its susceptibility towards elevated oxygen concentrations present already during the initial phase of the bioreactor cultivation, when a control strategy was chosen to keep pO_2_ values ≥30%.

To avoid high pO_2_ values during the initial phase of the cultivations, a different strategy for pO_2_ control was tested for the ∆*mshC* mutant, thanks to its susceptibility for elevated pO_2_ levels. By mixing air with nitrogen, pO_2_ in the bioreactor was adjusted to different levels at an initially constant stirring rate of 400 rpm. The results showed that, at a pO_2_ of max 20%, growth of the ∆*mshC* mutant proceeded with slightly reduced growth rate of 0.31 h^−1^ (Figure 1d) when compared with a growth rate of 0.37 h^−1^ (Figure 1c) for the WT. Moreover, at a pO_2_ of max 20%, *C. glutamicum* WT and ∆*mshC* mutant cells reached final optical densities of 48 and 41, respectively (Figure 1c,d).

To test for negative effects of elevated pO_2_ on growing cultures in bioreactors, *C. glutamicum* WT and the ∆*mshC* mutant were cultivated initially at a pO_2_ of max 20% for 3 h until optical densities of 7 and 6, respectively (Figure 1e,f). Subsequently, the pO_2_ was increased in a single step to 40%. Whereas growth of *C. glutamicum* WT continued after the increase of pO_2_ to a final OD of 28 in the course of cultivation (Figure 1e), growth of the ∆*mshC* mutant immediately stopped, resulting in a final OD of 9 (Figure 1f). These experiments showed that the MSH-deficient ∆*mshC* mutant is highly sensitive to pO_2_ levels above 20%. As MSH protects the cells against oxidative stress, these results indicate that, already at slightly increased pO_2_ levels, oxidative stress occurs during bioreactor cultivation.

### 3.2. Oxidation of the Mrx1-roGFP2 Biosensor Allows Monitoring the Changes in the MSH Redox Potential (E_MSH_) in C. glutamicum

**Figure 2:**
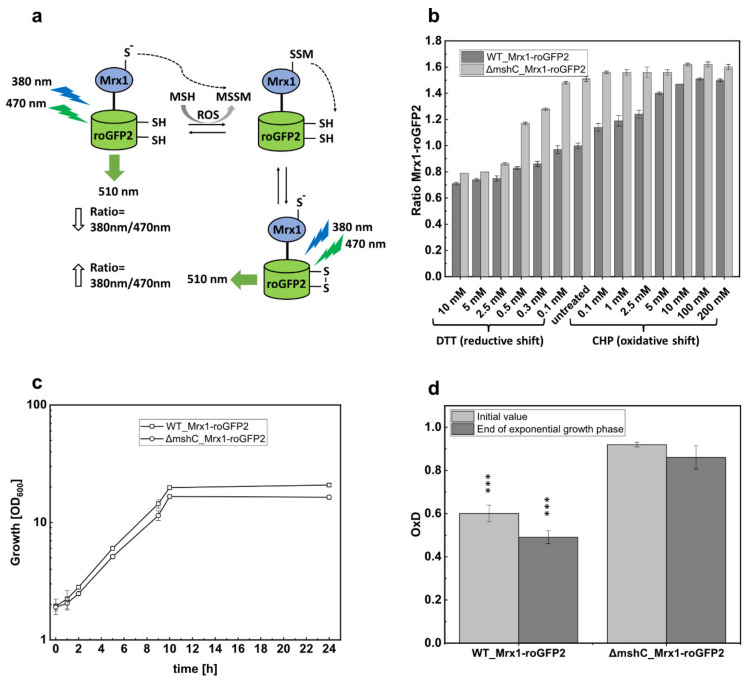
Schematic illustration of the Mrx1-roGFP2 biosensor response mechanism (**a**); the ratiometric Mrx1-roGFP2 biosensor response as shown by the 380/470 nm excitation ratio upon treatment with different concentrations of reductants (DTT) and oxidants (CHP) in *C. glutamicum* WT_Mrx1-roGFP2 (WT_Mrx1-roGFP2) and *C. glutamicum* ∆*mshC*_Mrx1-roGFP2 (Δ*mshC*_Mrx1-roGFP2) (**b**); shake flasks cultivations of WT_Mrx1-roGFP2 and Δ*mshC*_Mrx1-roGFP2 in 50 mL CGXII minimal medium (T = 30 °C, initial glucose concentration 15 g L^−1^, 150 rpm) (**c**); and biosensor oxidation degrees (OxD) derived from shake flask samples after inoculation and the end of exponential growth phase (**d**). Error bars indicate standard deviations from three independent experiments. OxD values were calculated by normalizing the samples to fully oxidized and reduced controls. OxD values of WT_Mrx1-roGFP2 are significantly different when compared with Δ*mshC*_Mrx1-roGFP2 OxD values at the *p* = 0.01 level (one-way analysis of variance (ANOVA) with Tukey´s test) ( *** *p* < 0.0001). MSH, mycothiol; ROS, reactive oxygen species; MSSM, mycothiol disulfide.

The observation that the MSH deficient ∆*mshC* mutant was impaired in growth with elevated oxygen concentrations prompted us to measure the changes in the MSH redox potential (*E*_MSH_) in *C. glutamicum* during bioreactor experiments. Thus, we applied the recently developed genetically encoded Mrx1-roGFP2 biosensor, which is stably integrated in the genome of *C. glutamicum* [29]. Redox sensitive GFP2 (roGFP2) harbors two Cys residues that form a disulfide bond upon oxidation, resulting in ratiometric changes of two excitation maxima in the fluorescence excitation spectrum [46]. Mrx1 further was shown to selectively reduce *S*-mycothiolated proteins as part of the Mrx1/MSH/Mtr electron pathway [19,47]. Moreover, the Mrx1-roGFP2 fusion was well characterized as a redox biosensor with respect to its selectivity towards MSSM in vitro [29]. Upon reaction with MSSM, the MSH moiety is transferred to Mrx1 and roGFP2, followed by intramolecular disulfide formation in roGFP2 and the concomitant change of its fluorescence excitation maxima [29] (Figure 2a). To define suitable settings for ratiometric fluorescence measurements, crude cell extracts of *C. glutamicum* WT_Mrx1-roGFP2 with integrated Mrx1-roGFP2 were prepared and treated with 10 mM DTT or 5 mM diamide for fully reduced and oxidized controls, respectively, as previously described [29]. The strongest fluorescence intensity alteration (emission intensity at 510 nm) under our settings was detected when the biosensor was excited at 380 nm and 470 nm (Figure S1). More specifically, upon oxidation of the probe, the excitation maximum at 380 nm increases with the subsequent decrease of the 470 nm excitation maximum, and vice versa upon reduction of the probe. Although the second fluorescence intensity maximum was described at 488 nm previously [29], this was out of the range of measurements of our available microplate reader. Thus, we used the calculation of the 380/470 nm fluorescence intensity ratios in our biosensor settings, which is an indicator of the MSH redox potential changes in *C. glutamicum*.

For in vivo determination of Mrx1-roGFP2 biosensor oxidation, *C. glutamicum* WT_Mrx1-roGFP2 and ∆*mshC*_Mrx1-roGFP2, both harboring the genome integrated biosensor Mrx1-roGFP2, were cultivated in shake flasks until the stationary phase was reached. Prior to fluorescence measurements, cells were harvested by centrifugation, washed twice with potassium phosphate buffer (100 mM; pH 7.0), and an optical density of 40 was adjusted as previously described [29]. Upon treatment with DTT and CHP, for reduction and oxidation of the biosensor probe, respectively, the 380/470 nm excitation ratio of Mrx1-roGFP2 was determined (Figure 2b). For non-treated samples, an equal volume of the respective buffer was added instead. Non-treated shake flask samples of *C. glutamicum* WT_Mrx1-roGFP2 and ∆*mshC*_Mrx1-roGFP2 revealed huge differences in terms of the biosensor oxidation ratio with 1.0 ± 0.02 and 1.52 ± 0.03, respectively (Figure 2b). However, the addition of DTT (reducing agent) or CHP (oxidizing agent) eliminated the biosensor ratio differences, resulting in fully oxidized and fully reduced biosensor ratios of 1.5–1.6 and 0.7–0.8, respectively (Figure 2b). As expected, growth of the mutant strain in shake flasks with minimal medium proceeded similar with a growth rate of 0.26 ± 0.02 h^−1^ when compared with the parental strain *C.glutamicum* WT_Mrx1-roGFP2 (0.27 ± 0.03 h^−1^) (Figure 2c). Biosensor measurements at the end of the exponential growth phase further revealed that the initial biosensor oxidation degrees (OxD; Equation (1)) were maintained highly oxidized (0.91 ± 0.01; 0.86 ± 0.05) in the MSH-deficient mutant and more reduced (0.6 ± 0.04; 0.49 ± 0.04) in the WT strain (Figure 2d).

This confirms a mycothiol redox potential (*E*_MSH_) (Equation (2)) in *C. glutamicum* WT_Mrx1-roGFP2 and ∆*mshC*_Mrx1-roGFP2 of −280 ± 2 mV and −255 ± 7 mV at the end of the exponential growth phase in shake flasks, respectively (Table 1). This observation is in accordance with the previous study, showing that *C. glutamicum* WT_Mrx1-roGFP2 maintains a highly reducing intracellular environment during the course of cultivation in shake flasks (−280–300 mV) [29]. In contrast, the MSH-deficient mutant showed a more oxidized intracellular environment [29]. It is likely that elevated ROS levels in the MSH mutant caused an oxidation of Mrx1-roGFP2. However, in contrast to bioreactor experiments with oxygen concentrations surpassing the air saturation (pO_2_ = 30%), growth of the MSH-deficient mutant was not impaired in shake flasks (Figure 2c), as seen for bioreactor cultivations with pO_2_ of 20% (Figure 1d), when compared with the WT strain. This indicates that ROS production under these conditions did not overwhelm ROS detoxification by MSH-independent enzymatic antioxidant systems in the ∆*mshC* mutant. The addition of the thiol-reactive oxidant NaOCl to shake flask cultures of the ∆*mshC* mutant was shown to be detrimental in terms of growth patterns [27], as observed during bioreactor experiments with oxygen concentrations surpassing the air saturation. This indicates the sensitivity of the ∆*mshC* mutant towards increased ROS production in bioreactors, supporting the role of MSH to overcome oxidative stress during fermentation. In contrast, MSH is not essential in aerobic shake flask cultures with lower oxygen tension, which is in agreement with the observed more reduced biosensor signals in the *C. glutamicum*_Mrx1-roGFP2 strain (Figure 2d) [29].

### 3.3. Mycothiol-Dependent Protection is Important when C. glutamicum is Exposed to Elevated Oxygen Concentrations

To investigate the oxidative response of the Mrx1-roGFP2 biosensor in *C. glutamicum* WT_Mrx1-roGFP2 at elevated oxygen concentrations, we performed bioreactor experiments with *C. glutamicum* WT_Mrx1-roGFP2 at a pO_2_ of ≥30%. The first fluorescence measurement revealed an almost fully oxidized biosensor.

**Figure 3:**
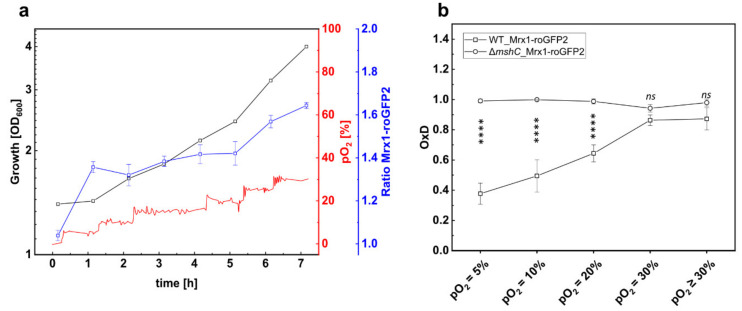
Growth and 380/470 nm excitation ratio of the Mrx1-roGFP2 biosensor of *C. glutamicum* WT_Mrx1-roGFP2 (WT_Mrx1-roGFP2) during batch cultivation in stirred bioreactors (**a**) and calculated oxidation degree (OxD) at different pO_2_ levels during batch fermentation conducted with WT_Mrx1-roGFP2 and the MSH-deficient mutant strain *C. glutamicum* Δ*mshC*_Mrx1-roGFP2 (Δ*mshC*_Mrx1-roGFP2) (**b**). Fermentation was performed in BIOSTAT^®^ B bioreactors using CGXII minimal medium (T = 30 °C, pH = 7.0; initial glucose concentration 20 g L^−1^). OxD values at respective pO_2_ levels were calculated by normalizing fluorescence measurements to fully oxidized (200 mM CHP, 15 min incubation) and reduced (10 mM DTT, 15 min incubation) controls. Error bars indicate standard deviation of six fluorescence measurements. Significance of difference between OxD values of WT_Mrx1-roGFP2 and Δ*mshC*_Mrx1-roGFP2 at different pO_2_ levels was determined by one-way ANOVA and Tukey´s test at the 0.01 level (^ns^ *p* ≥ 0.01, **** *p* < 0.00001).

To ensure that the biosensor response resulted from increased oxygen concentrations, the bioreactor cultivation of *C. glutamicum* WT_Mrx1-roGFP2 was performed with a stepwise pO_2_ gradient (Figure 3a). At the initial pO_2_ of 0% (set by providing 100% N_2_ as sole gas), the biosensor oxidation ratio was very low, indicating the presence of a reducing environment in the bioreactor at a pO_2_ of 0% (Figure 3a). Subsequently, the pO_2_ value was increased in 5% steps in the bioreactor and, at each of the pO_2_ steps, the signal ratio of the biosensor was determined 60 min after setting the pO_2_. As depicted in Figure 3a, the ratio of the biosensor increased when setting higher oxygen concentrations (pO_2_), indicating an oxidative stress response. At a pO_2_ of 30%, the biosensor oxidation degree (OxD) was determined as 0.86 ± 0.04 (Figure 3b), representing a highly oxidized environment in *C. glutamicum* WT under these conditions. Moreover, further increase of the pO_2_ value did not lead to enhanced OxD values, which are not significantly different to those determined for the MSH- deficient mutant strain (Figure 3b). In comparison, at lower pO_2_ values (pO_2_ = 5%; pO_2_ = 20%; pO_2_ = 25%), OxD values determined for the WT_Mrx1-roGFP2 strain were significantly lower than the fully oxidized biosensor probes for the Δ*mshC*_Mrx1-roGFP2 mutant strain (Figure 3b).

Notably, under aerobic conditions, the strongest oxidative shift occurred when surpassing the air saturation of 20%, resulting in an OxD shift of 0.33 from 0.53 ± 0.06 (pO_2_ = 20%) (which is in the range of OxD values determined for shake flask samples) towards highly oxidized values of 0.86 ± 0.04 (pO_2_ = 30%) (Figure 3b). This conforms an oxidative shift of *E*_MSH_ from −280 ± 6 mV (pO_2_ = 20%) to −256 ± 4 mV (pO_2_ = 30%) for the *C. glutamicum* WT_Mrx1-roGFP2 strain, whereas the redox potential of the mutant strain was highly oxidized at every tested pO_2_ level (Table 2). This oxidative shift is in agreement with the growth defect of the MSH-deficient mutant strain when surpassing the air saturation both when setting a constant pO_2_ = 30% (Figure 1d), but also with a stepwise increase of the pO_2_ value for the mutant strain harboring the redox biosensor Mrx1-roGFP2 (Figure S2).

Taken together, the strong oxidative shift of *C. glutamicum* WT_Mrx1-roGFP2 in bioreactor cultivations indicates the requirement of the non-enzymatic antioxidant and LMW thiol MSH to overcome oxidative stress when the oxygen concentration surpasses the air saturation.

## 4. Discussion

Utilization of respiratory chains for aerobic metabolism comes along with the generation of ROS [6,9]. To eliminate these toxic byproducts, aerobic organisms developed antioxidant defense mechanisms including enzymatic and non-enzymatic protection systems [12,13,14,15,16,17]. The abundant LMW thiol MSH functions to maintain the reduced state of the cytoplasm and represents the main non-enzymatic antioxidant in high-GC Gram-positive bacteria, such as the industrial platform organism *C. glutamicum* [19,27,48]. Apart from MSH, *C. glutamicum* encodes highly efficient enzymatic detoxification systems, such as the superoxide dismutase (SOD) [49] and methionine sulfoxide reductases (Msr) [50]; catalase (KatA) and the peroxiredoxins mycothiol peroxidase (Mpx) [15,51]; and thiol-peroxidase (Tpx) [27]. The metalloenzyme superoxide dismutase (SOD) (EC 1.15.1.1) catalyzes the dismutation of superoxide anions to H_2_O_2_ and oxygen. An *E. coli sodA sodB* double mutant was impaired in growth during batch cultivation when the dissolved oxygen concentration was shifted from 30% to 300% air saturation, indicating its importance in ROS detoxification during bioreactor experiments [52]. H_2_O_2_ is subsequently converted to H_2_O and O_2_ via the H_2_O_2_ scavenging systems KatA, Mpx, and Tpx in *C. glutamicum* [15,27]. This avoids a further conversion to the highly toxic hydroxyl radical.

In contrast to KatA, the peroxiredoxins Mpx and Tpx employ peroxidatic Cys residues for H_2_O_2_ detoxification, leading to *S*-mycothiolation and intramolecular disulfides of Mpx and Tpx. Regeneration or their catalytic activities requires coupling to either the Trx/TrxR or Mrx1/MSH/Mtr pathway, whereas in biochemical studies, the latter was shown to be kinetically favored under oxidative stress [14,15,47,53]. Moreover, the Trx/TrxR pathway is known to be employed by many biochemical systems for regeneration, and thus regarded as rate limited [14]. Thus, the Mrx1/MSH/Mtr pathway functions in de-mycothiolation under stress conditions, when Trx/TrxR is busy with reduction of cellular disulfides. In *C. glutamicum*, the *katA* mutant was shown to be highly sensitive towards H_2_O_2_ treatment, resulting in a strong oxidation of the Mrx1-roGFP2 biosensor [29]. In contrast, no sensitivity towards H_2_O_2_ exposure was observed in the *mpx* and *tpx* mutants, which showed similar biosensor responses under H_2_O_2_ stress as the wild type [29]. *C. glutamicum* WT was shown to be resistant towards 100 mM H_2_O_2_ and the Mrx1-roGFP2 biosensor did not respond to 10 mM H_2_O_2_ [29]. Of note, 1–5 mM H_2_O_2_ resulted in a maximal roGFP2 biosensor oxidation in *E. coli* [12]. This indicates that KatA plays a crucial role for H_2_O_2_ detoxification, whereas the contribution of Mpx and Tpx is neglectable.

Notably, KatA of *C. glutamicum* possesses a remarkably high activity and is even commercially available (Merck, CAS Number 9001-05-2). Despite the extraordinary enzymatic detoxification power of KatA, elevated oxygen concentrations during batch fermentations resulted in cell death of the MSH-deficient *C. glutamicum* mutant. ROS production rates are proportional to the collision frequency of oxygen and redox enzymes [9]. Consequently, the rate of ROS production inside the cell directly depends on the oxygen concentration in the extracellular environment [7,9]. This indicates that ROS production in bioreactor cultivations overwhelmed the remaining antioxidant systems and that MSH as an additional antioxidant is required to provide protection against oxidative stress at elevated oxygen concentrations. Consistently, a strong oxidative response of the Mrx1-roGFP2 biosensor was observed when *C. glutamicum* WT was exposed to oxygen concentrations that were shown to be harmful for the MSH-deficient mutant strain in bioreactors. This confirms the requirement of MSH as supporting antioxidant and, consequently, an oxidative redox shift of the 2MSH/MSSM redox couple occurring in *C. glutamicum* WT strains under these conditions. Thus, the increased MSH synthesis or the overexpression of Mtr for MSH recycling could be beneficial to improve the intracellular production process, providing a promising strategy for the development of highly robust industrial production strains.

MSH has multiple antioxidant functions by scavenging free radicals either directly or as a cofactor for antioxidant enzymes [19]. When the oxygen concentration surpasses the air saturation, MSH becomes a crucial player to overcome oxidative stress, despite the presence of other highly efficient enzymatic antioxidant systems working independently of MSH.

## Figures and Tables

**Table 1:** Mycothiol (MSH) redox potential (*E*_MSH_; Nernst equation) during shake flask cultivations.

Shake Flask	*E*_MSH_ (mV)	
	WT_Mrx1-roGFP2 ^(a)^	ΔmshC_Mrx1-roGFP2 ^(b)^
Initial value	−274 ± 2	−248 ± 2
End of exponential growth phase	−280 ± 2	−255 ± 7

^(a)^*C. glutamicum* WT harboring the redox biosensor Mrx1-roGFP2. ^(b)^ MSH-deficient mutant of *C. glutamicum* harboring the redox biosensor Mrx1-roGFP2.

**Table 2:** Mycothiol redox potential (*E*_MSH_; Nernst equation) during bioreactor cultivations.

Bioreactor pO_2_ level (%)	*E*_MSH_ (mV)	
	WT_Mrx1-roGFP2 ^(a)^	ΔmshC_Mrx1-roGFP2 ^(b)^
5	−287 ± 4	−204 ± 2
20	−280 ± 6	−191 ± 2
25	−272 ± 3	−208 ± 22
30%	−256 ± 4	−242 ± 7
≥30%	−246 ± 28	−218 ± 23

^(a)^*C. glutamicum* WT harboring the redox biosensor Mrx1-roGFP2. ^(b)^ MSH-deficient mutant of *C. glutamicum* harboring the redox biosensor Mrx1-roGFP2.

## Supplementary Materials

The following supplementary figures are available online at https://www.mdpi.com/2076-3921/9/10/969/s1, Figure S1: Spectral scan of the Mrx1-roGFP2 biosensor of crude cell extracts of *C. glutamicum* WT_Mrx1-roGFP2; Figure S2: Growth and oxidation ratio of the biosensor Mrx1-roGFP2 of *C. glutamicum* ∆mshC_Mrx1-roGFP2 during batch cultivation in stirred bioreactors.
