# Supplementary material for: The Industrial Organism Corynebacterium glutamicum Requires Mycothiol as Antioxidant to Resist Against Oxidative Stress in Bioreactor Cultivations
Source: Antioxidants (Basel). 2020 Oct 9;9(10):969. doi: 10.3390/antiox9100969 (PMC7599745; doi:10.3390/antiox9100969)
Supplement: Supplementary file 1 [file antioxidants-09-00969-s001.pdf]

Supplementary Material to the Manuscript:

The industrial organism *Corynebacterium glutamicum* requires mycothiol as antioxidant to resist against oxidative stress in bioreactor cultivations

By Fabian Stefan Franz Hartmann, Lina Clermont, Quach Ngoc Tung, Haike Antelmann, and Gerd Michael Seibold

Correspondence: gesei@dtu.dk

The supplementary material comprises two figures: Figure S1: Spectral scan of the Mrx1-roGFP2 biosensor of crude cell extracts of *C. glutamicum* WT_Mrx1-roGFP2, Figure S2: Growth and oxidation ratio of the biosensor Mrx1-roGFP2 of *C. glutamicum* ΔmshC_Mrx1-roGFP2 during batch cultivation in stirred bioreactors.

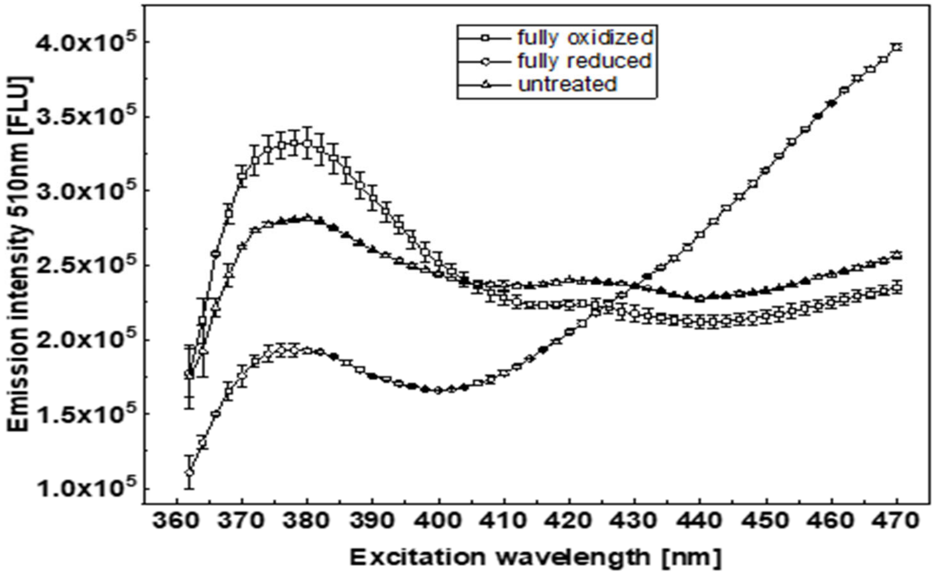

Figure S1. The ratiometric response of the Mrx1-roGFP2 biosensor upon treatment with 10 mM DTT or 5 mM diamide of crude cell extracts of *C. glutamicum* WT_Mrx1-roGFP2. Prior the preparation of cell extracts, cells were cultivated in 2xTY complex medium until the stationary phase. Cells were harvested, washed twice with 100 mM potassium phosphate buffer (pH= 7.0) and crude cell extracts prepared using a Ribolyzer (6000 rpm, 30 second, 4 cycles). Prior fluorescence analysis, samples were incubated for 15 min. to allow a full oxidation or reduction of the biosensor probe. While the first excitation maximum of Mrx1-roGFP2 is observed at 380 nm, the second excitation maximum is displayed at 470 nm with a respective emission wavelength of 510nm due to the limited bandwidth of the spectrophotometer. Measurements exceeding an excitation wavelength of 470 nm would lead to inaccurate fluorescence measurements. Thus, biosensor measurements were limited by setting the second excitation maximum to 470 nm.

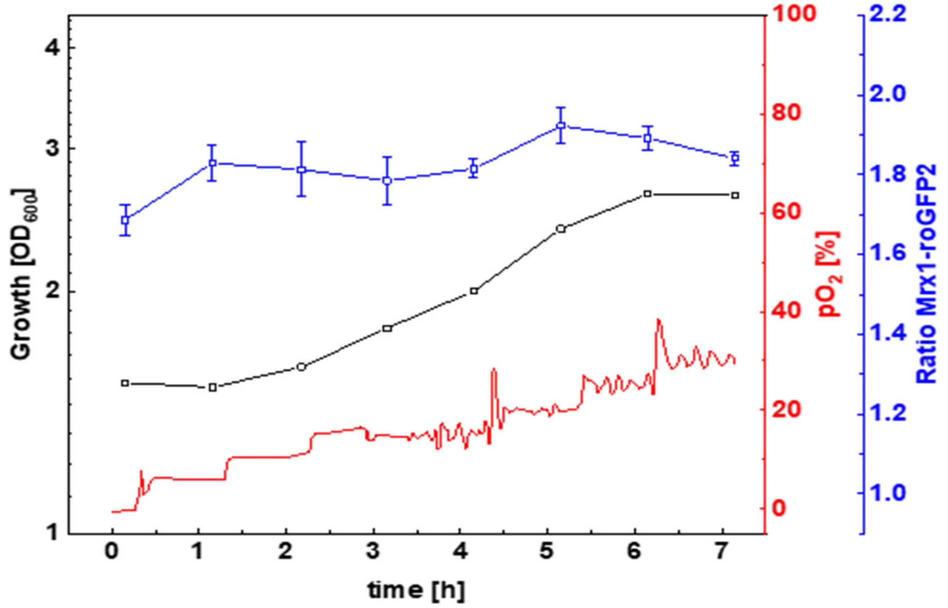

Figure S2. Growth and the 380/470 nm excitation ratio of the Mrx1-roGFP2 biosensor of *C. glutamicum* Δ*mshC*_Mrx1-roGFP2 during batch cultivation in stirred bioreactors. Cultivation was performed in CGXII minimal medium (T= 30

°C, pH= 7.0; initial glucose concentration 20 g L^{-1}). To allow monitoring of the biosensor response, batch cultivation was initially started with pO_2=0% by flooding the bioreactor with 100% nitrogen. Subsequently, pO_2 values were stepwise increased by 5% and samples taken after 60 minutes of incubation. For fluorescence measurements, cells were harvested, centrifuged and finally an optical density of 40 adjusted using 100 mM potassium phosphate buffer (pH= 7.0). One representative batch cultivation is shown. A fully oxidized biosensor at pO_2=5% was detected in two independent experiments. Growth was monitored by measuring the optical density at 600nm. Fermentations were performed in BIOSTAT® B bioreactors. Data were collected with the software MFCS. Fluorescence measurements were conducted in replicates (n=6).
